# A Fiber Bragg Grating Based Torsional Vibration Sensor for Rotating Machinery

**DOI:** 10.3390/s18082669

**Published:** 2018-08-14

**Authors:** Jingjing Wang, Li Wei, Ruiya Li, Qin Liu, Lingling Yu

**Affiliations:** School of Mechanical and Electronic Engineering, Wuhan University of Technology, Wuhan 430070, China; jingjingwangwhut@163.com (J.W.); liruiya@whut.edu.cn (R.L.); m17786416229@163.com (Q.L.); 18883278903@163.com (L.Y.)

**Keywords:** fiber Bragg grating, torsional vibration, rotating machinery

## Abstract

This paper proposes a new type of torsional vibration sensor based on fiber Bragg grating (FBG). The sensor has two mass ball optical fiber systems. The optical fiber is directly treated as an elastomer and a mass ball is fixed in the middle of the fiber in each mass ball fiber system, which is advantageously small, lightweight, and has anti-electromagnetic interference properties. The torsional vibration signal can be calculated by the four FBGs’ wavelength shifts, which are caused by mass balls. The difference in the two sets of mass ball optical fiber systems achieves anti-horizontal vibration and anti-temperature interference. The principle and model of the sensor, as well as numerical analysis and structural parameter design, are introduced. The experimental conclusions show that the minimum torsional natural frequency of the sensor is 27.35 Hz and the torsional vibration measurement sensitivity is 0.3603 pm/(rad/s^2^).

## 1. Introduction

Rotating machinery has become an important branch of mechanical equipment and is widely used in industrial production. Mechanical vibration often results in various malfunctions in rotating machines during operation. The vibration of rotating machinery can be classified into bending vibration, axial vibration and three forms of torsional vibration. Axial vibration and bending vibration are easier to detect due to the obvious forms of vibration, so many more mature detection methods are available for these vibration types after a long period of research. Conversely, torsional vibration form is not obvious, the detection is complication and initially has not received enough attention. Torsional vibration of shafts is an important form of vibration in all kinds of rotary machinery. Torsional vibration is circumferential vibration caused by torque changing with time acting on the shaft [1]. Long-term torsional vibration can cause shaft structure stress fatigue failure when the vibration frequency is close to the natural frequency of the structure, resulting in greater fracture. Therefore, studying methods and techniques of detecting torsional vibration of rotating machinery is of considerable significance.

Many scholars have contributed to the study of torsional vibration measurements. As early as 1916, German scientist Geiger designed a mechanical torsional vibration meter to measure torsional vibration of engine shafting, which marked the beginning of torsional vibration measurement. After a century of deep research, measurement methods for torsional vibration are being constantly enriched, and mainly include contact methods, non-contact methods, and sensorless methods [1].

The contact methods are a class of methods that directly measure torsional vibration by using the sensor rotating with the rotating shaft. Ji et al. [2] proposed measuring torque changes in shafts with two resistance strain gauges attached to the surface of the shaft, which were 45° to the axis and perpendicular to each other. Yang et al. [3] used two identical piezoelectric acceleration sensors mounted symmetrically in the circumferential tangential direction on the rotating shaft to measure the tangential acceleration of the rotating shaft. Liao et al. [4] presented a torsional vibration measuring device based on the principle of inertia, effectively eliminating radial vibration. Binglin et al. [5] proposed a novel and highly accurate indirect torsional vibration receptance measurement method for shaft structures.

Non-contact methods involve sensors being fixed outside rotating shafting that do not rotate with the shaft. Sometimes, this method uses the existing structure or auxiliary structures are installed on the shaft. Jiang et al. [6] proposed a measuring method for the torsional vibration of aeroengine rotors by adopting eddy current displacement sensors and structures on the aeroengine rotor, such as aeroengine speed measurement fluted discs. He et al. [7] improved the above method [6] to avoid the impact on the measurements caused by profile error. They used groove or protrusion on the shaft as the key phase mark and recorded the key phase signal measured by a non-contact displacement sensor as the reference starting point. Zhao et al. [8] presented a torsional vibration measurement system consisting of a photoelectric incremental encoder and a signal acquisition instrument. Zhang et al. [9] described a method for rotor torsional vibration measurement based on the electromagnetic induction effect. This method is accurate, simple to install, and is user-friendly in terms of operation.

Furthermore, there are some non-contact measurement methods based on the laser Doppler principle [10,11,12,13]. Xiang et al. [10] proposed a laser torsional vibrameter to measure the torsion vibration of a rotating shaft system under electrical network impact, which was based on laser Doppler velocimetry. Huang et al. [11] introduced a measurement method for the torsional vibration of high-speed rotary machine, which was more real-time, and the dynamic range was considerably extended compared with other measurement methods based on the laser Doppler technique. Liu et al. [12] developed two types of fiber-optic sensor based on the laser Doppler principle for instantaneous torsional vibration measurements: either differential or reference instruments, which were more easily applicable.

In addition to the above methods, sensorless detection techniques have been used for the measurement of torsional vibration, in which conventional physical quantity sensors are no longer used, but the stator current of the drive motor is directly extracted and analyzed to monitor the working condition of the rotating equipment and the motor itself [14,15,16]. Shi et al. [14] used sensorless detection techniques to carry out a vibration monitoring experiment and verified the feasibility of the stator current signal of the motor to measure the vibration fault, which was more sensitive to torsional vibration than transverse vibration.

Among the above torsional vibration measurement methods, electrical measurement and magnetoelectric measurement methods are susceptible to electromagnetic interference, non-contact gear measuring methods have trouble transmitting signals, and the laser Doppler method uses expensive equipment and the measurement process is susceptible to environmental interference.

Compared with the above sensing technology, the emerging fiber grating sensors have many advantages including their small size, immunity from electromagnetic interference, strong environmental adaptability, easy implementation of dynamic and distributed detection, and long-distance transmission. Fiber Bragg grating (FBG) has been applied to the measurement of torsional vibration. Sheng et al. [15,16] designed a two FBG rotary position sensor based system to detect the rotating angle of a rotor. The two FBGs are affixed to the metal rod along the axial direction and separated from each other by 90 degrees on the circumference. When the rotor rotates, the force of the two magnets on the rotor to the magnet at the end of the metal rod constantly changes, which causes the deflection curve of the metal rod to change continuously, thus resulting in the constant change in wavelength of the FBGs. Due to the use of a magnetic field, the sensor is susceptible to external interference. Yu et al. [17] designed a device for measuring static and tiny torsion. The inner ring is fixed on the base, and one end of the two FBGs is affixed on the surface of the rotatable outer ring, and the other end is fixed on the base. The sensor is not affected by temperature and has a sensitivity of 0.743 nm/°. However, it is not suitable for torsional measurement of dynamic rotating machines. Kruger et al. [18] stuck two FBGs on opposite sides of the shaft in the direction of 45 ° along the axis to measure torsional vibration. On this basis, Li et al. [19] established a complete strain sensing model and analyzed the decoupling principle of bending and torsional coupled vibrations, which was verified by experiments on a rotor platform. The theory of this measurement method is based on the equal diameter circular axis, so it is not applicable to stepped axes. In addition, this method requires FBGs to be directly affixed to the shaft, so it is not suitable for severe working conditions.

In order to overcome the above mentioned drawbacks, a new type of torsional vibration sensor based on fiber Bragg grating is proposed in this paper. The sensor has two mass ball fiber systems. A mass ball is fixed in the middle of the fiber in each mass ball fiber system. This system is small, lightweight, and has anti-electromagnetic interference properties. The torsional vibration signal can be calculated by the FBGs’ wavelength shifts that are caused by the mass ball. The combination of the two sets of mass ball optical fiber systems effectively resists transverse vibration and temperature interference. 

## 2. Sensor Principles and Model 

According to the sensing principle of FBG, when the fiber grating receives axial stretching or compression, or the ambient temperature changes, the center wavelength of the fiber grating correspondingly shifts due to the grating cycle and the effective refractive index changes. The relation between strain, temperature, and center wavelength shifts Δ*λ* can be written as [20]:

(1) $$\frac{\Delta \lambda }{\lambda }=(1-P_{e})\Delta \varepsilon +(\alpha_{f}+\xi_{f})\Delta t$$

where *P_e_* is the strain-optic coefficient of optical fiber, *a_f_* is the coefficient of thermal expansion, and *x_f_* is the thermal-optic coefficient. Normally, *P_e_* is 0.22, *a_f_* is 0.55 × 10^−6^/°C, and *x_f_* is 5.775 × 10^−6^/°C.

### 2.1. Principle of the Sensor

**Figure 1:**
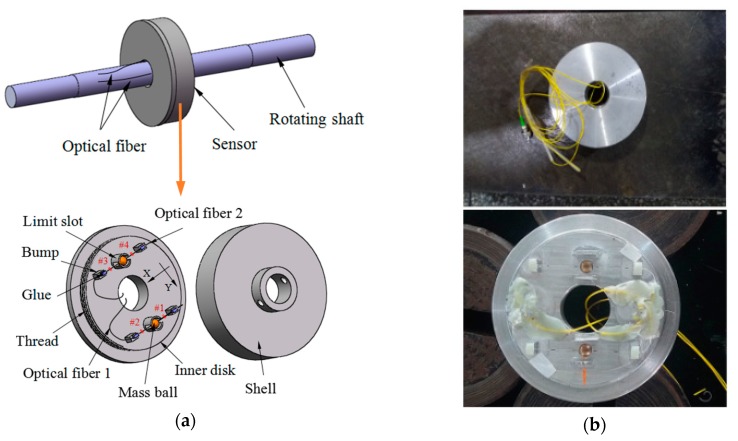
(**a**) The schematic diagram and (**b**) photograph of the proposed rotating mechanical torsional vibration sensor.

A schematic diagram and photograph of the proposed rotating mechanical torsional vibration sensor are shown in Figure 1. The sensor is mainly composed of a shell, an inner disk, two copper mass balls, and four FBGs. The shell and the inner disk are connected together by a thread, and each disk has a center hole. The center hole of the shell was fitted with a rotating shaft, the diameter of which was smaller than that of the center hole of the inner disk. Each of two identical copper mass balls was provided with a through hole, of which the diameter was slightly larger than that of the optical fiber to allow the optical fiber to pass through the mass ball. Two mass balls were fixed on the middle of optical fiber 1 and optical fiber 2 with 502 glue. #1FBG and #2FBG were placed on both sides of the mass ball on optical fiber 1, #3FBG and #4FBG were placed on both sides of the mass ball on optical fiber 2. The same tension was applied to optical fiber 1 and optical fiber 2, two sides of which were mounted on the bumps of the inner disk with glue. The two optical fibers were parallel to each other and the distances to the center of the round hole were equal. There were two limit slots on the inner disk used to limit the movement range of the two mass balls to prevent optical fibers from being pulled off.

As shown in Figure 1, the sensor is mounted on the rotating shaft, the optical fiber are drawn from the gap between the inner disk of the sensor and the rotating shaft. When torsional vibration of the rotating shaft occurs, the forces in the tangential of the two mass balls change. The relationship between torsional vibration acceleration and the strain of the fiber grating can be obtained by establishing the differential relation between the forces acting on the two mass balls. Finally, the torsional vibration signal can be measured by the wavelength shift of the four FBGs.

### 2.2. Mathmatical Model of the Sensor

**Figure 2:**
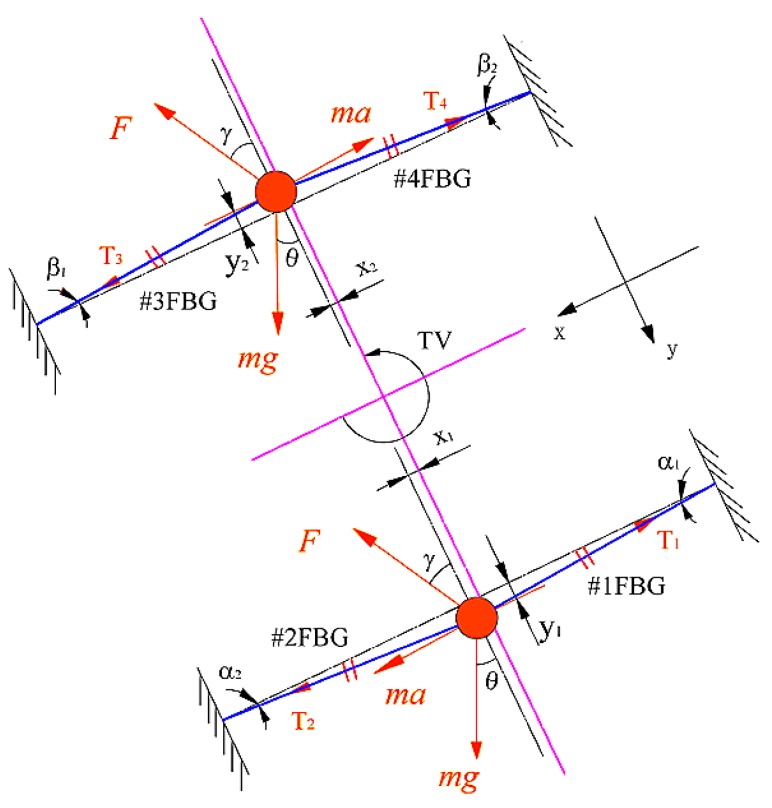
The force diagram of the mass ball optical fiber systems of the sensor.

**Figure 3:**
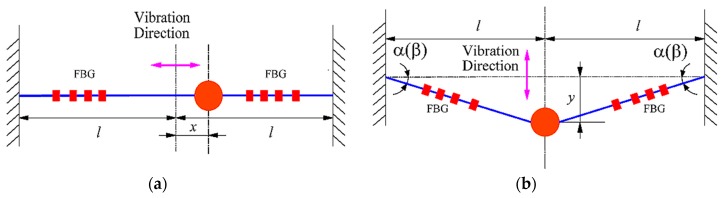
Equivalent vibration model of sensor’s mass ball optical fiber system: (**a**) axial vibration model along the *X* direction and (**b**) lateral vibration model along the *Y* direction.

The sensor rotates with the rotating shaft and is subjected to torsional vibration in a clockwise direction. The force diagram of the sensor is shown in Figure 2. The vibration of the sensor′s mass ball optical fiber system can be seen as a combination of its axial vibration along the *X* direction (as shown in Figure 3a) and the lateral vibration along the *Y* direction (as shown in Figure 3b). The forces acting on the mass ball can be divided into several categories: (1) gravity *mg*, the direction of which is always vertical downward, where *m* is mass of the mass ball and *g* is the acceleration of gravity; (2) the inertia force -*ma* caused by torsional vibration, the direction is tangential along the rotating shaft, where *a* is the acceleration of mass ball caused by torsional vibration; (3) interference force *F*, which is the inertia force caused by the radial vibration of the rotating shaft; and (4) the pulling force of the optical fiber, of which the direction is outward along the fiber.

Initially, the same tension *T*_0_ is applied to the two fibers to make #1FBG, #2FBG, #3FBG, and #4FBG have an equal pre-strain *ε*_0_. The relationship between tension *T*_0_ and pre-strain *ε*_0_ is:

(2) $$T_{0}=EA\varepsilon_{0}$$

where *E* represents the Young′s Modulus of optical fiber and *A* represents the cross-sectional area of the optic fiber. Since the mass balls are fixed on the fiber, the mass balls are subjected to the first three forces to create a slight displacement in the *X* and *Y* direction, causing the fibers to stretch or shrink, and so the mass balls are subjected to the fourth force simultaneously. According to the balance of force and the law of orthogonal decomposition, we can determine the relation between the first three forces and strain of the fibers.

Firstly, we analyzed the forces of the mass balls and the strain of FBGs in the *X* direction of the sensor. For optical fiber 1, we determined from Figure 2 that the first three forces of the mass ball in the *X* direction are *mg*sin *θ*, *ma*, and *F*sin *γ*, which make the mass ball move *x_1_* in the *X* direction. As a result, #1FBG is stretched and #2FBG is compressed, and the strain increments of #1FBG and #2FBG are Δε*_x_*, and −Δε*_x_*. So, Equation (3) is obtained for optical fiber 1 in the *X* direction,

(3) $$mg\sin\theta +ma+F\sin\gamma =2EA\Delta \varepsilon_{x}$$

where *θ* is the angle between mg and *Y* axis direction, and *γ* is the angle between *F* and *Y* axis direction.

Using a similar process, we can determine from Figure 2 that # FBG is stretched and #4FBG is compressed in the *X* direction, the strain increments of #3FBG and #4FBG are Δ*ε_x_*′ and −Δ*ε_x_*′, respectively. So Equation (4) for optical fiber 2 in the *X* axis direction can be expressed as:

(4) $$mg\sin\theta -ma+F\sin\gamma =2EA\Delta \varepsilon_{x}^{\prime }$$

Figure 3a depicts the axial (*X*-direction) vibration model of the mass ball fiber grating system of the sensor. The axial stiffness of the fiber can be written as:

(5) $$k_{f}=\frac{EA}{l}$$

where *l* represents half of the initial length of the optical fiber. The axial equivalent stiffness of the mass ball fiber grating system can be expressed as:

(6) $$K_{x}=2k_{f}=\frac{2EA}{l}$$

So the resonant frequency of the axial vibration of the mass ball-fiber grating system *ω_x_* can be expressed as:

(7) $$\omega_{x}=\sqrt{\frac{K_{x}}{m}}=\sqrt{\frac{2EA}{ml}}$$

Then, we analyzed the forces of the mass balls and the strain of the FBGs in the *Y* direction of the sensor. As shown in Figure 2 for optical fiber 1, the first three forces acting on the mass ball in the *Y* axis direction are *mg*cos*θ*, 0, and *F*cos*γ*, which makes the mass ball fiber system vibrate laterally, and the displacement of the mass ball in the *Y* direction is *y_1_*. #1FBG and #2FBG are stretched in the *Y* direction, which increases the tension of optical fiber 1 by Δ*T*. The angle between the optical fibers on both sides of the mass ball and the *X* direction are both *a*. The strain increases of #1FBG and #2FBG are both Δ*ε_y_*, so we can obtain Equation (8) as:

(8) $$mg\cos\theta -F\cos\gamma -2\Delta T\sin\alpha =mr\omega^{2}$$

where *ω* is angular velocity of rotational axis and *r* is the distance from the position of the mass ball to the axis.

Similarly, for optical fiber 2, the tension increment of optical fiber 2 is Δ*T*′, the angle between the optical fibers on both sides of the mass ball and the *X* direction are both *β*, and the strain increments of #3FBG and #4FBG are both Δε*_y_*. We can obtain Equation (9) as:

(9) $$-mg\cos\theta +F\cos\gamma -2\Delta T^{\prime }\sin\beta =mr\omega^{2}$$

In the transverse vibration model shown in Figure 3b, the system freely vibrates when the resultant force of the above three kinds of changing external forces acting on the mass ball is kept at zero. In the position shown in the picture, the displacement of the mass ball is *y*, and the tension of the fiber can be described as:

(10) $$T=T_{0}+k_{f}(\sqrt{l^{2}+y^{2}}-l)$$

The resultant force of the tensions of the optical fibers on both sides of the mass ball can be written as:

(11) $$F_{y}=2T\sin\alpha$$

The lateral equivalent stiffness of the mass ball-fiber grating system can be expressed as:

(12) $$K_{y}=\frac{F_{y}}{y}$$

Combining Equations (5) and (10)–(12), we can obtain Equation (13). Since *y* is much smaller than *l*, Equation (13) is further simplified as:

(13) $$K_{y}=\frac{2T_{0}+2k_{f}(\sqrt{l^{2}+y^{2}}-l)}{\sqrt{l^{2}+y^{2}}}\approx \frac{2T_{0}}{l}$$

So, according to the theory of vibration and Equation (2), the resonant frequency of the lateral vibration of the mass ball-fiber grating system can be expressed as:

(14) $$\omega_{y}=\sqrt{\frac{2T_{0}}{ml}}=\sqrt{\frac{2EA\varepsilon_{0}}{ml}}$$

Combining Equations (3) and (4), the relationship between torsional vibration acceleration and the strain increments of the optical fibers in the X direction can be described as:

(15) $$2ma=2EA(\Delta \varepsilon_{x}-\Delta \varepsilon_{x}^{\prime })$$

where *ε*_1_, *ε*_2_, *ε*_3_ and *ε*_4_ are the strains of 1#FBG, 2#FBG, 3#FBG, and 4#FBG, respectively, which can be expressed by:

(16) $$\left\{ \begin{array}{l} \varepsilon_{1}=\Delta \varepsilon_{y}+\Delta \varepsilon_{x}+\varepsilon_{0}\\ \varepsilon_{2}=\Delta \varepsilon_{y}-\Delta \varepsilon_{x}+\varepsilon_{0}\\ \varepsilon_{3}=\Delta \varepsilon_{y}^{\prime }-\Delta \varepsilon_{x}^{\prime }+\varepsilon_{0}\\ \varepsilon_{4}=\Delta \varepsilon_{y}^{\prime }+\Delta \varepsilon_{x}^{\prime }+\varepsilon_{0} \end{array}\right.$$

According to Equation (1), we can obtain the following equation:

(17) $$\begin{array}{l} \frac{\Delta \lambda_{1}}{\lambda }=(1-P_{e})\varepsilon_{1}+(\alpha_{f}+\xi_{f})\Delta t\\ \frac{\Delta \lambda_{2}}{\lambda }=(1-P_{e})\varepsilon_{2}+(\alpha_{f}+\xi_{f})\Delta t\\ \frac{\Delta \lambda_{3}}{\lambda }=(1-P_{e})\varepsilon_{3}+(\alpha_{f}+\xi_{f})\Delta t\\ \frac{\Delta \lambda_{4}}{\lambda }=(1-P_{e})\varepsilon_{4}+(\alpha_{f}+\xi_{f})\Delta t \end{array}$$

Combining Equations (16) and (17), the strain increment Δ*ε_x_* of optical fiber 1 and the strain increment Δ*ε_x_*′ of optical fiber 2 in the *X* direction can be expressed as:

(18) $$\left\{ \begin{array}{l} \Delta \varepsilon_{x}=\frac{1}{2(1-P_{e})}(\frac{\Delta \lambda_{1}}{\lambda_{1}}-\frac{\Delta \lambda_{2}}{\lambda_{2}})\\ \Delta \varepsilon_{x}^{\prime }=\frac{1}{2(1-P_{e})}(\frac{\Delta \lambda_{4}}{\lambda_{4}}-\frac{\Delta \lambda_{3}}{\lambda_{3}}) \end{array}\right.$$

where *λ*_1_, *λ*_2_, *λ*_3,_ and *λ*_4_ represent the center wavelength of #1FBG, #2FBG, #3FBG, and #4FBG, respectively; and Δ*λ*_1_, Δ*λ*_2_, Δ*λ*_3,_ and Δ*λ*_4_ represent the center wavelength shift of #1FBG, #2FBG, #3FBG and #4FBG, respectively.

Since *λ*_1_ ≈ *λ*_2_ ≈ *λ*_3_ ≈ *λ*_4_ >> Δ*λ*_1_, Δ*λ*_2_, Δ*λ*_3_, Δ*λ*_4_, and combining Equations (15) and (18), the torsional vibration acceleration a of the mass ball can be written as:

(19) $$a=\frac{EA}{2m(1-P_{e})}\frac{\Delta \lambda_{1}+\Delta \lambda_{3}-\Delta \lambda_{2}-\Delta \lambda_{4}}{\lambda }$$

According to the relationship between angular acceleration *β* and tangential acceleration *a* expressed as *a* = *βr*, the relationship between angular acceleration *β* and FBGs’ wavelength shift is described as:

(20) $$\beta =\frac{EA(\Delta \lambda_{1}+\Delta \lambda_{3}-\Delta \lambda_{2}-\Delta \lambda_{4})}{2mr(1-P_{e})\lambda }$$

So the angular acceleration sensitivity of the sensor can be expressed as:

(21) $$S=\frac{\Delta \lambda_{1}+\Delta \lambda_{3}-\Delta \lambda_{2}-\Delta \lambda_{4}}{\beta }=\frac{2mr(1-P_{e})\lambda }{EA}$$

It can be known from Equation (21) that the angular acceleration sensitivity *S* is a certain value, which is related to the mass *m* of the sensor′s mass ball and radius *r* of the mass ball′s location. Therefore, the desired sensitivity can be obtained by adjusting these two parameters.

## 3. Numerical Analysis and Structural Parameter Design

From Equations (7) and (14), we can see that the mass ball optical fiber vibration system of the sensor has two natural frequencies: *ω_y_* and *ω_x_*. In order to study the vibration characteristics and measurement range of the sensor, it was necessary to determine the minimum natural frequency of the sensor. We know that *ω_y_* << *ω_x_*, so the minimum natural frequency of the torsional vibration of the sensor is *ω_y_*, which is affected by *m*, *l*, and ε*_0_*. According to Equation (21), the sensitivity is affected by *m* and *r*.

**Figure 4:**
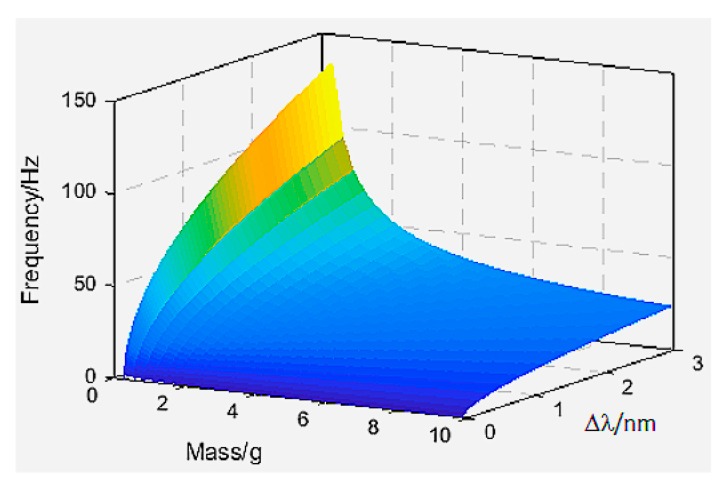
The relationship between frequency *ω_y_* and mass and center wavelength shift of fiber grating under prestrain.

Since the minimum natural frequency is related to *l*, and the sensitivity is independent of it, we firstly determined the value of *l*. The smaller value of *l* should be taken as far as possible because the minimum natural frequency is negatively correlated with *l*. The length of fiber grating gate used in the experiment was 5 mm, and the possible maximum radius of the mass ball was 13 mm (according to the mass range), so *l* was taken as 20 mm. The Young’s Modulus of optical fiber *E_f_* is 69 GPa and the outer diameter of the optic fiber was 125 μm. So, according to Equation (14), the relationship between frequency *ω_y_* and mass (*m*)/center wavelength shift (Δ*λ*) of fiber grating under prestrain *ε_0_* is shown in Figure 4.

It can be seen from Figure 4 that the natural frequency *ω_y_* decreases with increasing mass of the mass ball, and increases with increasing center wavelength shift of FBG under prestrain. When the mass of the mass ball was small, Δ*λ* had a more obvious impact on the natural frequency. Thus, the value of *m* and Δ*λ* were set according to the vibration range to be measured by the sensor. In order to obtain as large a vibration range as possible, a small–medium mass and a large Δ*λ* should be selected.

**Figure 5:**
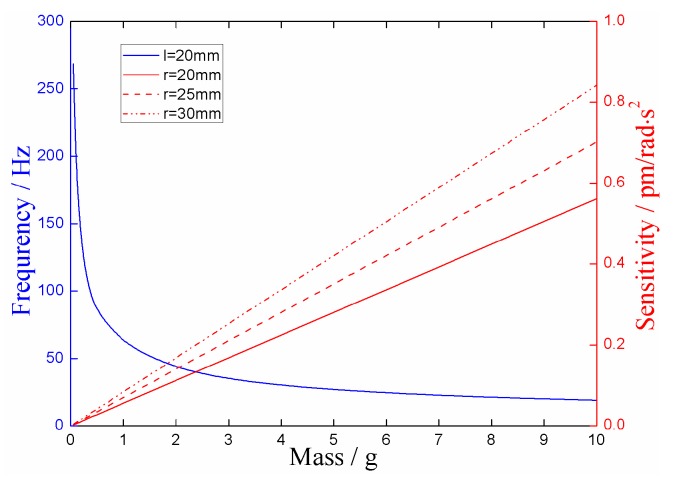
The curves of the sensor’s frequency and sensitivity change with mass of the mass ball.

*ω_y_* and *S* are related to the mass of mass ball. When Δ*λ* = 2 nm, *l* = 20 mm, according to Equations (14) and (21). The curves of the natural frequency and sensitivity versus mass are shown in Figure 5. From Figure 5, we can see that as the mass of the mass ball increases, the minimum natural frequency of the system decreases non-linearily. When *m* > 2 g, the natural frequency curve becomes flat and the change in mass has little effect on the minimum natural frequency. However, the sensitivity of the system increases linearly with the increase in the mass of the mass ball; the higher the value of *r*, the higher the value of the corresponding sensitivity.

In summary, in order to simultaneously ensure that the sensitivity is large enough and the natural frequency is not too small, we should choose a mass ball with a moderate range of mass. In this paper, the mass of the mass ball was 4.08 g, half the length of the optical fiber *l* was 20 mm, and the distance from the position of the mass ball to the axis *r* was 30 mm.

**Figure 6:**
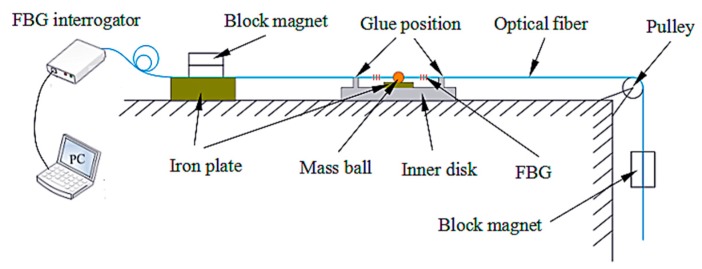
Schematic diagram of the pre-strain loading device of the sensor’s fiber Bragg gratings (FBGs).

Figure 6 is schematic diagram of the pre-strain loading device of the sensor′s FBGs. The left end of the optical fiber is pressed on the iron plate by magnet blocks to fix it, and the drawn optical fiber is connected to the FBG interrogator (resolution: 1 pm; sampling rate: 4000 Hz; test bandwidth: 1525–1565 nm) and the computer. The right end of the optical fiber is clamped by magnet blocks after being passed around the pulley. The mass ball is supported by the iron plate to ensure that the mass ball has no force on the optical fiber when the optical fiber is applied with tension in the assembly process of the sensor. In the optical fiber binding process of the sensor, the number of right-side magnet blocks can be adjusted to change the tensile force of the optical fiber on the basis of wavelength shift of the sensor’s FBG obtained by the computer. Four FBGs are distributed on two grating strings, each of which has two FBGs. The fiber Bragg grating used in the sensor was single-mode fiber grating, fiber type SMF-28e, and SLSR ≥ 4 dB. The gate area of each FBG was 5 mm, the emission spectral bandwidth was 0.1–0.2 nm, the reflectivity was 80%, and the core diameter was 125 mm. When the wavelength shift of sensor’s FBG reached 2 nm, the weight of the suspended magnet blocks was 150 g, so the optical fiber was subjected to a pull force of 1.5 N, and the glue was loaded at this time. When the colloid solidified, the center wavelength of each FBG of the sensor shifted, as shown in Table 1. The average FBGs center wavelength shift of the two mass ball optical fiber systems were 1.999 nm and 2.032 nm. According to Equation (14), the torsional vibration natural frequencies of the sensor’s #1 and #2 mass ball optical fiber systems were 29.99 and 29.74 Hz, respectively. The sensitivity of the sensor was 0.3603 pm/(rad/s^2^), which was calculated by Equation (21).

## 4. Experiments and Discussion

**Figure 7:**
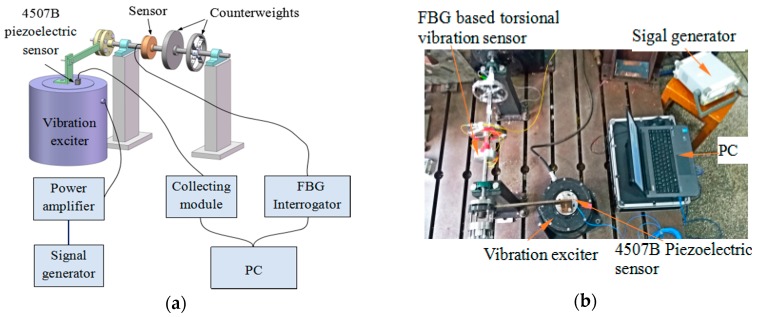
(**a**) The schematic diagram and (**b**) photograph of sensing characteristics experiments for the FBG-based torsional vibration sensor.

Figure 7 shows a schematic diagram and photograph of the sensing characteristics experiment for the FBG-based torsional vibration sensor. The sensor and two counterweights are mounted on the rotary shaft, which is fitted with bearings at both ends. One end of the beam is fixed to the end of the shaft by means of bolts and couplings, and the other end is connected with the vibration exciter through a connecting piece. Thus, the vibration exciter provides a tangential vibration signal to the shaft through the beam. The vibration exciter is driven by a signal generator and power amplifier; the signal generator generates a control signal, which is transmitted to the exciter after amplification by the power amplifier, and the exciter outputs the corresponding excitation signal. A 4507B piezoelectric sensor is fixed at the vibration exciter, the signal of which is transmitted to the computer via the collecting module, so we can record the magnitude of the acceleration of the excitation signal from the piezoelectric acceleration sensor. The optical fiber output interface of the FBG torsional vibration sensor is connected to FBG interrogator, which is connected to the computer on which the FBG signal is displayed.

### 4.1. Amplitude-Frequency Property Experiments

#### 4.1.1. Exciter Excitation Experiments

**Figure 8:**
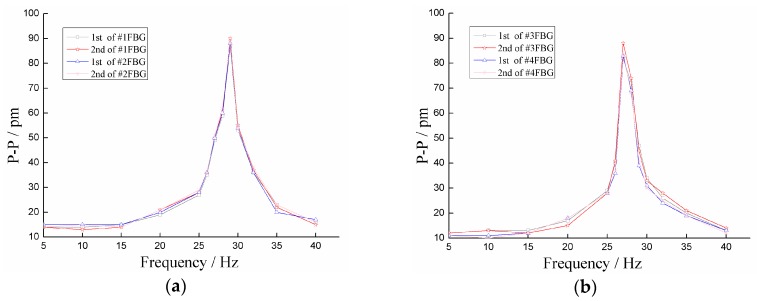
(**a**) Amplitude-frequency curves of #1 mass ball optical fiber system. (**b**) Amplitude-frequency curves of #2 mass ball optical fiber system.

Firstly, we studied the sensor’s amplitude-frequency properties. In the experimental process, the acceleration amplitude was set at 5 m/s^2^ and remained unchanged. The frequency changed from 0 to 1000 Hz. The experiment was repeated two times. The amplitude-frequency characteristic curves of the sensor’s two mass ball-optical fiber systems are shown in Figure 8. From Figure 8, we determined: (1) the amplitude-frequency curves of the two mass ball optical fiber systems are roughly the same, and when the frequency is within 0 and 15 Hz, the curve is almost parallel to the horizontal axis, which indicates that the working range of the sensor is 0–15 Hz; (2) the resonant frequencies of the two mass ball optical fiber systems of the sensor were 29 and 27 Hz, which are basically consistent with the numerical simulation resonant frequencies of 29.99 and 29.74 Hz. Moreover, the resonant frequency of the #1 mass ball optical fiber system was slightly larger than the resonant frequency of the #2 mass ball optical fiber system, which may be caused by the deviation in the position of the two systems’ mass balls during assembly.

#### 4.1.2. Hammering Excitation Experiments

**Figure 9:**
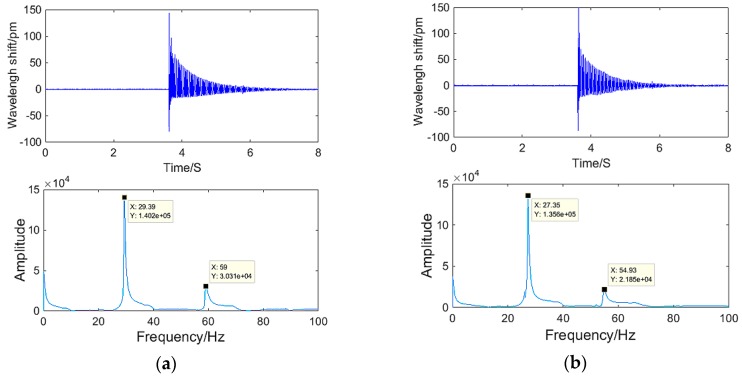
Time domain and spectrum map of (**a**) #1 mass ball and (**b**) #2 mass ball optical fiber systems.

In order to further explore and verify the dynamic characteristics of the sensor, we carried out hammering excitation experiments on the sensor. As shown in Figure 7, the signal generator and power amplifier are switched off, the exciter does not work and only plays the role of supporting the beam. Using the hammer to hit the end of the beam in the vertical direction, a tangential pulse signal was provided to the shaft and then the shaft was free to vibrate. The torsional vibration resonant frequency was obtained from the response signals of the sensor. Figure 9 shows the time domain and spectrum map of the response signals of the sensor under hammering excitation. It can be seen from Figure 9 that the responses of the two mass ball optical fiber systems of the sensor are good, and the first-order torsional vibration natural frequency of the sensor′s #1 and #2 mass ball optical fiber systems are 29.39 and 27.35 Hz, respectively, which is more accurate than the result of the exciter excitation experiment and consistent with the theoretical value.

### 4.2. Sensitivity Experiments

In the experimental system shown in Figure 7, the exciter provides the torsional vibration signal for the shaft, the beam is in the horizontal position, and the distance between the excitation point on the beam and the axis of the shaft *d* is 305 mm. If the excitation acceleration of the exciter is *a*, the torsional vibration acceleration *β* would be *a/d*. During the experiment, the frequency of excitation acceleration was always kept at 2 Hz, the excitation acceleration amplitude increased from 5 to 30 m/s^2^, and then decreased to 5 m/s^2^ with the purpose of investigating the hysteresis of the sensor. The experimental data were recorded every 5 m/s^2^, and the experiment was repeated three times.

**Figure 10:**
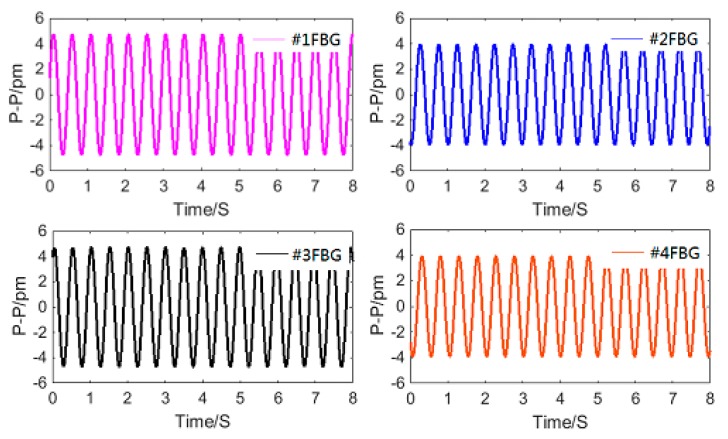
Time domain signal of each FBG under the excitation with acceleration amplitude of 20 m/s^2^ and frequency of 2 Hz.

**Figure 11:**
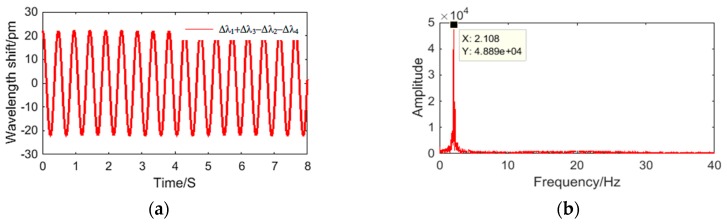
(**a**) Time domain and (**b**) spectrum map of Δ*λ*_1_ + Δ*λ*_3_ − Δ*λ*_2_ − Δ*λ*_4_ under the excitation with acceleration amplitude of 20 m/s^2^ and frequency of 2 Hz.

**Figure 12:**
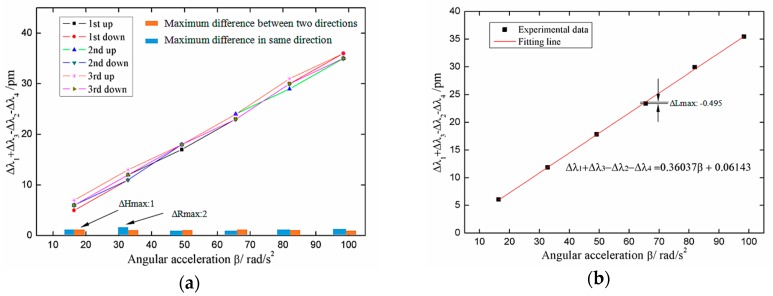
The relationship between Δ*λ*_1_ + Δ*λ*_3_ − Δ*λ*_2_ − Δ*λ*_4_ and angle acceleration. (**a**) The plot and (**b**) the linear fitted curve of Δ*λ*_1_ + Δ*λ*_3_ − Δ*λ*_2_ − Δ*λ*_4_ versus vibration angle acceleration.

Figure 10 shows the time domain signal of each FBG under excitation with an acceleration amplitude of 20 m/s^2^ and frequency of 2 Hz. The signals of #1FBG and #4FBG had the same phase and change trend, and the signals of #2FBG and #3FBG had the same phase and change trend. According to Equation (21) and as shown in Figure 11, we can obtain the change curve of Δ*λ*_1_ + Δ*λ*_3_ − Δ*λ*_2_ − Δ*λ*_4_ under the above excitation. It can be seen from Figure 11 that the frequency of Δ*λ*_1_ + Δ*λ*_3_ − Δ*λ*_2_ − Δ*λ*_4_ is basically consistent with the excitation frequency. Thus, the plot of Δ*λ*_1_ + Δ*λ*_3_ − Δ*λ*_2_ − Δ*λ*_4_ versus vibration angle acceleration is shown in Figure 12. Figure 12a shows the maximum difference in the measured data between each experiment in the same or two directions. The average value of the upper limit of Δ*λ*_1_ + Δ*λ*_3_ − Δ*λ*_2_ − Δ*λ*_4_ in the experiment Δ*λ* max was 36 pm, thus the repeatability error and hysteresis error of the sensor can be calculated as ΔRmax/Δ*λ*max = 5.556%, and ΔHmax/Δ*λ*max = 2.778%, respectively. In order to further study the sensor characteristics of the sensor, we averaged the six sets of experimental data and obtained the linear fitted curve shown in Figure 12b. We obtained the following data from Figure 12b: (1) the maximum difference between the measured value and the fitted straight line ΔLmax was 0.495, so the linearity of the sensor ΔLmax/Δ*λ* max was 1.376%; (2) the fitting equation can be expressed as Δ*λ*_1_ + Δ*λ*_3_ − Δ*λ*_2_ − Δ*λ*_4_ = 0.3604*β* + 0.0614, so according to Equation (21), the angular acceleration sensitivity of the sensor was 0.3604 pm/(rad/s^2^). The average single FBG sensitivity was 0.3604/4 pm/(rad/s^2^), considering the wavelength shift of each FBG under the prestress (Table 1) and the wavelength shift of each FBG caused by the vibration interference (which can reach the level of gravitational acceleration). For measuring the rotation, the safety factor was chosen as 100 (one level above the gravitational acceleration), so the measurement range was 1994/100/(0.3604/4) rad/s^2^ = 221 rad/s^2^.

### 4.3. Anti-Interference Characteristic Experiments

**Figure 13:**
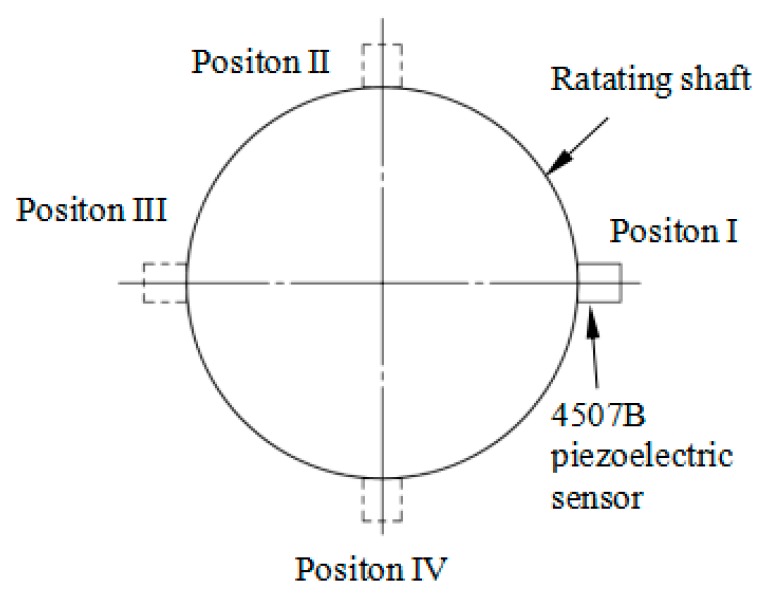
Paste position diagram of 4507B piezoelectric acceleration sensor.

In the actual measurement of the torsional vibration of a shaft, the lateral vibration of the shaft inevitably simultaneously occurs with the torsional vibration, so we needed to explore the sensor’s anti-interference characteristics on lateral vibration. We slightly modified the experimental apparatus in Figure 7 by attaching a 4507B piezoelectric acceleration sensor to the surface of the rotating shaft. We hammered the rotating shaft in the measuring direction of the 4507B piezoelectric sensor (perpendicular to the paste surface of the sensor) to provide the shaft with a transverse vibration signal, and acceleration of hammering should meet the condition *a < g* = 9.8 m/s^2^. Then the sensor’s paste position was changed according to Figure 13 and the above steps were repeated.

**Figure 14:**
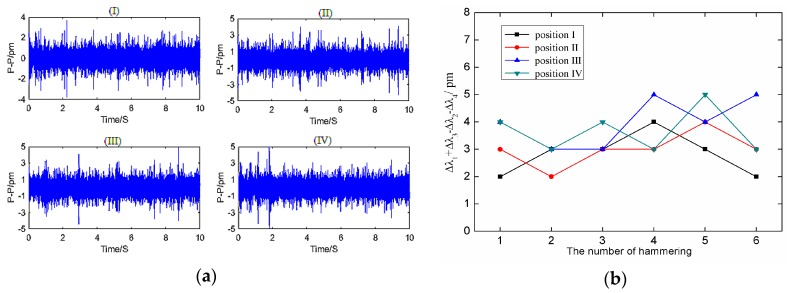
The results of anti-interference characteristic experiments. (**a**) Time-domain plots of Δ*λ*_1_ + Δ*λ*_3_ − Δ*λ*_2_ − Δ*λ*_4_ at four different hammering locations. (**b**) The results of Δ*λ*_1_ + Δ*λ*_3_ − Δ*λ*_2_ − Δ*λ*_4_ at different percussion positions under multiple beats.

The time-domain waveforms of FBGs obtained from this experiment were similar to those in Figure 9. According to Equation (21), the time-domain plots of Δ*λ*_1_ + Δ*λ*_3_ − Δ*λ*_2_ − Δ*λ*_4_ and Δ*λ*_1_ + Δ*λ*_3_ − Δ*λ*_2_ − Δ*λ*_4_ shown in Figure 14a at four different hammering positions can be obtained. Figure 14b shows the results of Δ*λ*_1_ + Δ*λ*_3_ − Δ*λ*_2_ − Δ*λ*_4_ at different percussion positions under multiple beats. It can be seen that Δ*λ*_1_ + Δ*λ*_3_ − Δ*λ*_2_ − Δ*λ*_4_ changes in the range of 3 pm at different percussion positions, and there is no significant difference between them, which indicates that the sensor has good anti-jamming characteristics for lateral vibration.

### 4.4. Temperature Effects Experiments

**Figure 15:**
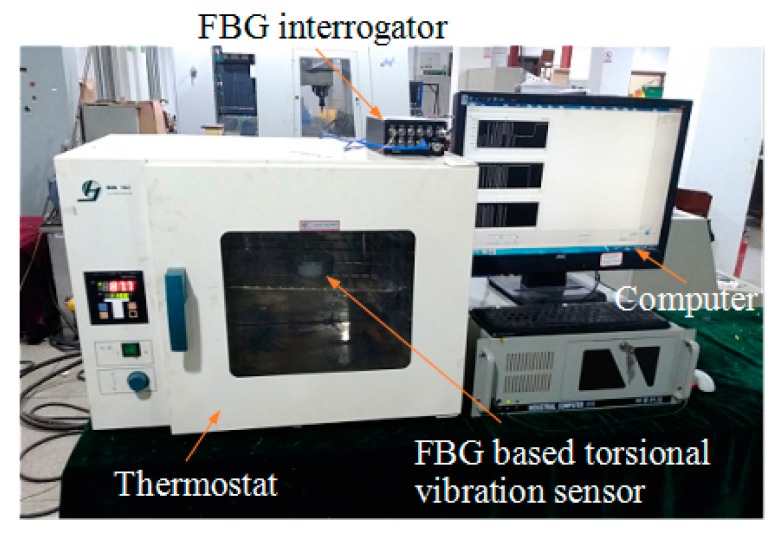
Temperature influence testing system.

**Figure 16:**
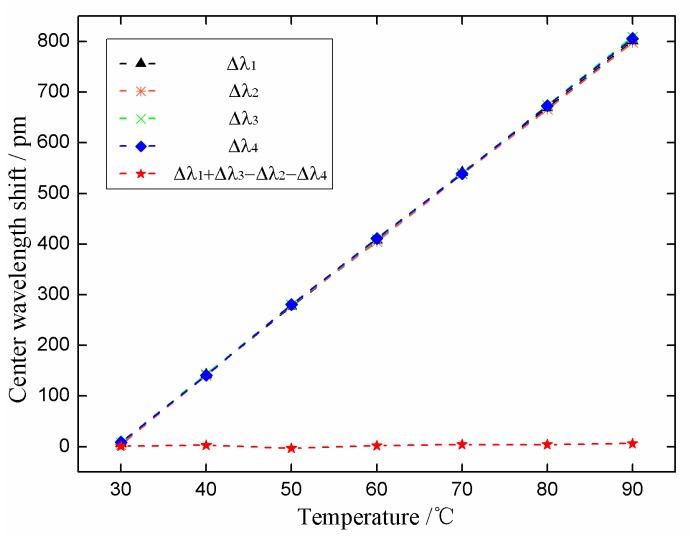
Temperature response curves of this sensor.

From Equations (17) and (18), we can see that the proposed sensor can compensate for temperature, which means the measurement results are not disturbed by temperature. In order to verify this characteristic, we examined the temperature influences on the FBGs of the sensor. Figure 15 shows the temperature influence testing system. A thermostat was used to control the surrounding temperature of the sensor. The experiments were performed within the range of 30 to 90 °C and the sampling internal was 10 °C. After averaging three sets of experimental data, the temperature response curves of this sensor were created (Figure 16). The figure shows that the center wavelength shift of each FBG varied linearly versus temperature, and the temperature response Δλ_1_ + Δλ_3_ − Δλ_2_ − Δλ_4_ of this sensor was insensitive and had little variation from −3 to 6 pm in the range of 30 to 90 °C, which indicates that temperature interference have been effectively compensated.

## 5. Conclusions

A new type of torsional vibration sensor based on fiber Bragg grating was proposed in this paper. The sensor has two mass ball optical fiber systems. In each mass ball fiber system, the optical fiber was directly treated as an elastomer and a mass ball was fixed in the middle of the optical fiber. The torsional vibration signal was calculated by the wavelength shifts of four FBGs, which were caused by the mass balls. The difference in the two sets of mass ball optical fiber systems achieved anti-horizontal vibration and anti-temperature interference. Moreover, this sensor is small, lightweight and provides anti-electromagnetic interference. Experimental results showed that the minimum torsional natural frequency of the sensor is 27.35 Hz and the torsional vibration measurement sensitivity is 0.3603 pm/(rad/s^2^). In addition, the mass of the sensor, the mass of the sphere, and the length of the fiber can be adjusted according to different requirements to obtain better dynamic performance to measure the torsional vibration in different ranges.

## Figures and Tables

**Table 1:** Center wavelength shift of each fiber Bragg grating (FBG) of the sensor.

Number of FBGs	#1FBG	#2FBG	#3FBG	#4FBG
Initial center wavelength (nm)	1539.688	1542.635	1549.608	1551.632
Wavelength after prestress (nm)	1541.682	1544.638	1551.639	1553.665
Wavelength shift after prestress (nm)	1.994	2.003	2.031	2.033

## References

[1] Ye J., Li T., Zeng Z.L. (2014). Review of Research on Shafts’ Torsional Vibration Measurement. Ship Eng..

[2] Ji T.G., Ding L.B., Zhang H. (2003). Wireless sensor data acquisition system of rotating component. J. Transducer Technol..

[3] Yang Z.J., Liao M.F. (2008). Measurement of Torsional Vibration using Acceleration Sensors. Noise Vib. Control.

[4] Liao M.F., Duan S.G., Li Y.F. (2006). New Method to Measure the Torsional Vibration of Rotor. Acta Aeronaut..

[5] Lv B.L., Ouyang H.J., Li W.Y., Shuai Z., Wang G. (2016). An indirect torsional vibration receptance measurement method for shaft structures. J. Sound Vib..

[6] Jiang Y., Liao M., Wang S. (2013). New measuring method for torsional vibration of aeroengine rotor. J. Vib. Meas. Diagn..

[7] He Q., Du D. (2007). Study on intelligent measurement system of torsional vibration for turbine-generator shafts. Chin. J. Sci. Instrum..

[8] Zhao W.S., Xiong S.B. (2006). Application of Hilbert Transform for Measurement of Torsional Vibration. J. Taiyuan Univ. Technol..

[9] Zhang M.J., Guo D.A. (2009). Method of Torsional Vibration Measurement Based on Electromagnetic Induction. J. Exp. Mech..

[10] Xiang L., Yang S., Gan C. (2012). Torsional vibration measurements on rotating shaft system using laser doppler vibrometer. Opt. Lasers Eng..

[11] Huang Z., Liu B., Dong Q. (2006). Research on the torsional vibration measurement based on laser doppler technique. Acta Opt. Sin..

[12] Liu T.Y., Berwick M., Jackson D.A. (1992). Novel fiber-optic torsional vibrometers. Rev. Sci. Instrum..

[13] Miles T.J., Lucas M., Halliwell N.A., Rothberg S. (1999). Torsional And Bending Vibration Measurement On Rotors Using Laser Technology. J. Sound Vib..

[14] Shi X.J., Shao J.P., Si J.S., Li B.N. Experiment and simulation of rotor’s torsional vibration based on sensorless detection technology. Proceedings of the IEEE International Conference on Automation and Logistics.

[15] Sheng H.J., Tsai P.T., Lee W.Y., Lin G.R., Sun H.T. (2012). Random rotary position sensor based on fiber Bragg gratings. IEEE Sens. J..

[16] Sheng H.J., Lin G.R., Tsai P.T., Yang C.A., Kuo M.H., Sun H.T., Fu M.Y., Liu M.F. Random-rotational angle sensor based on fiber Bragg gratings. Proceedings of the 17th Opto-Electronics and Communications Conference.

[17] Yu H., Yang X., Tong Z., Cao Y., Zhang A. (2011). Temperature independent rotational angle sensor based on fiber Bragg grating. IEEE Sens. J..

[18] Kruger L., Swart P.L., Chtcherbakov A.A., van Wyk A.J. (2004). Non-contact torsion sensor using fibre Bragg gratings. Meas. Sci. Technol..

[19] Li T.L., Shi C.Y., Tan Y.G., Zhong Z.D. (2007). Fiber Bragg Grating Sensing-Based Online Torque Detection on Coupled Bending and Torsional Vibration of Rotating Shaft. IEEE Sens. J..

[20] Hill K.O., Meltz G. (1997). Fiber bragg grating technology fundamentals and overview. J. Light. Technol..

